# Bone-CNN: A Lightweight Deep Learning Architecture for Multi-Class Classification of Primary Bone Tumours in Radiographs

**DOI:** 10.3390/biomedicines14020299

**Published:** 2026-01-29

**Authors:** Behnam Kiani Kalejahi, Sajid Khan, Rakhim Zakirov

**Affiliations:** 1 Computer Science Department, School of Engineering, Central Asian University, Tashkent 111221, Uzbekistan; s.khan@centralasian.uz; 2 Medical School, Central Asian University, Tashkent 111221, Uzbekistan; r.zakirov@centralasian.uz

**Keywords:** primary bone tumours, radiograph classification, deep learning, lightweight CNN architecture, bone-CNN

## Abstract

**Background/Objectives:** Accurate classification of primary bone tumors from radiographic images is essential for early diagnosis, appropriate treatment planning, and informed clinical decision-making. While deep convolutional neural networks (CNNs) have shown strong performance in medical image analysis, their high computational complexity often limits real-world clinical deployment. This study aims to develop a lightweight yet highly accurate model for multi-class bone tumor classification. **Methods:** We propose **Bone-CNN**, a computationally efficient CNN architecture specifically designed for radiograph-based classification of primary bone tumors. The model was evaluated using the publicly available Figshare Radiograph Dataset of Primary Bone Tumors, which includes nine distinct tumor classes ranging from benign to malignant lesions and originates from multiple imaging centres. Performance was assessed through extensive experiments and compared against established baseline models, including DenseNet121, EfficientNet-B0, and MobileNetV2. **Results:** Bone-CNN achieved a test accuracy of 96.52% and a macro-AUC of 0.9989, outperforming all baseline architectures. Both quantitative and qualitative evaluations, including confusion matrices and ROC curve analyses, demonstrated robust and reliable discrimination between challenging tumor subtypes. **Conclusions:** The results indicate that Bone-CNN offers an excellent balance between accuracy and computational efficiency. Its strong performance and lightweight design highlight its suitability for clinical deployment, supporting effective and scalable radiograph-based assessment of primary bone tumors.

## 1. Introduction

Deep learning has recently been applied extensively to the diagnosis of primary bone tumours on radiographs and multimodal imaging. Wang et al. proposed CNN models to distinguish primary bone tumours from bone infections on radiographs with high diagnostic accuracy by only using relatively heavy architectures and binary tasks [1]. Von Schacky et al. introduced multitask deep learning for simultaneous detection, segmentation, and classification of primary bone tumours on radiographs [2], while Song et al. leveraged incomplete multimodal X-ray/CT/MRI data to improve tumour classification [3]. Several multicentre studies have demonstrated that deep learning can reliably detect or differentiate bone tumours around the knee joint [4,5] and even support radiologists in histological subtype classification [6].

Recent advances in medical image analysis further demonstrate the growing role of deep learning in extracting clinically meaningful patterns from complex biomedical data. Transformer-based and hybrid architectures have shown strong performance in challenging segmentation and classification tasks, such as dental plaque segmentation under unconstrained conditions [7] and collaborative instrument segmentation in minimally invasive surgery [8]. These studies highlight the capacity of modern deep learning models to generalise across imaging conditions, but they also reveal a trend toward increasingly complex architectures that may be impractical for routine clinical deployment, particularly in radiography-based workflows.

Primary bone tumours represent a heterogeneous group of benign and malignant lesions with distinct prognostic and therapeutic implications. Accurate subtype identification is critical for guiding biopsy, surgical planning, and referral to oncology services. Radiographs remain the first-line imaging modality in many settings due to accessibility and cost-effectiveness, yet interpretation is challenging because of overlapping morphological features among tumour subtypes. While prior AI studies have focused on binary tasks, real-world decision-making often requires distinguishing multiple categories, similarly to recent efforts in musculoskeletal disorders, such as tendinopathy classification [9]. This complexity underscores the need for robust multi-class models that can support radiologists in triaging cases and optimising treatment pathways.

In parallel, progress in other medical imaging domains, such as cardiac imaging, ultrasound microvessel analysis, and signal-based diagnostics, demonstrates that task-specific network design can significantly reduce computational burden without sacrificing performance. For example, lightweight deep learning frameworks have enabled fast denoising and super-resolution in ultrasound imaging [10,11], as well as efficient heart sound classification through multichannel feature fusion [12]. These advances suggest that carefully optimised architectures can achieve clinically viable performance while remaining computationally efficient: an insight that is particularly relevant for radiograph-based bone tumour analysis.

Most of these models employ large backbones, such as ResNet, DenseNet or custom multi-branch networks [1,2,3,6,13]. They achieve strong accuracy at the cost of high parameter counts and inference time, which limits real-time deployment, especially in settings where radiographs are the first and sometimes only imaging modality. Reviews on AI in bone and soft-tissue tumours similarly conclude that many published models are computationally demanding and insufficiently optimised for clinical workflow integration [14,15,16,17,18].

Although the existing CNN-based approaches for bone tumour analysis rely on the mentioned architectures, such as DenseNet or ResNet, and produce promising performances, usage of these architectures results in high memory consumption due to their complexities, as well as resulting in longer inference times. These limitations pose challenges in real-time deployment in clinical devices with low processing capabilities.

Furthermore, the heterogeneity of bone lesions has been highlighted in recent pathology studies [19], reinforcing the importance of accurate subtype differentiation for clinical decision-making. Molecular and immunological investigations also emphasise that distinct tumour subtypes may exhibit markedly different biological behaviours and treatment responses, further motivating precise preoperative classification [8,20]. Inspired by this body of work, Bone-CNN is designed as a lightweight, radiograph-specific architecture that preserves high multi-class performance while keeping the parameter budget and FLOPs substantially below commonly used backbones such as DenseNet121, EfficientNet-B0 [13,14], and advanced architectures such as ResNet50-CBAM and CNNT [21]. It is evaluated on the public Figshare dataset of primary bone tumours released by Yao et al. [22], which provides a standardised benchmark for classification, localisation, and segmentation.

## 2. Related Works

### 2.1. Deep Learning for Primary Bone Tumour Imaging

Over the last few years, there has been a rapid increase in deep learning applications for primary bone tumours across multiple imaging modalities. Radiograph-based approaches are the most relevant to our work.

Wang et al. developed CNN models to distinguish tumours from infections on radiographs, achieving high binary classification performance [1]. Von Schacky et al. proposed a multitask deep learning framework performing detection, segmentation, and classification of primary bone tumours on radiographs [2], but such designs are computationally heavy and complex.

Song et al. extended the problem to incomplete multimodal imaging, using deep learning to integrate X-ray, CT, and MRI data for improved tumour classification [3]. While multimodal fusion improved performance in cases where multiple imaging sequences were available, this approach assumes access to advanced imaging that is not always present in first-line settings. Xu et al. and Shao et al. confirmed CNN reliability for knee tumour detection in multi-centre cohorts [4,5]. Xie et al. showed that CNNs can assist radiologists in histological classification [6]. Li et al. and Breden et al. focused on detection/localisation using YOLO and CNNs [23,24], while Ye et al. extended multitask designs to MRI [13]. Reviews highlight gaps in transparency, efficiency, and clinical readiness [14,15,16,17,18].

In contrast, Bone-CNN uses a single, widely available modality (radiographs), which is a lightweight architecture optimised for efficiency, and it targets nine-way classification on a public multi-centre dataset.

### 2.2. Musculoskeletal Radiograph AI and Fracture Detection as a Foundation

The development of Bone-CNN is also informed by broader advances in AI for musculoskeletal (MSK) radiographs. The MURA dataset and associated CNN models by Rajpurkar et al. demonstrated that deep learning can reach near-radiologist-level performance in abnormality detection across multiple MSK regions [25]. Saif et al. introduced capsule networks for abnormality detection in musculoskeletal radiographs [26], exploring alternative architectures beyond standard CNNs.

A substantial body of work has focused on fracture detection, which shares similar challenges with bone tumour analysis [27,28,29,30,31,32].

Bone-CNN builds on this foundation by adopting depthwise separable convolutions and a multi-scale feature aggregation strategy, similar in spirit to efficient architectures used in fracture detection [27,29,32], while tailoring the design to the morphological characteristics of primary bone tumours.

### 2.3. Datasets and Benchmarks for Primary Bone Tumour AI

Yao et al. published a large, curated multi-centre radiograph dataset with nine tumour classes [22]. Our work uses this dataset for training and evaluation, ensuring reproducibility and external validity, unlike prior studies that were limited to subsets or narrow differential diagnoses.

### 2.4. Methodological Gaps and Our Contributions

Across these prior studies, three main methodological trends emerge:

1. Heavy backbones and complex multi-task designs.

Many models achieve excellent performance but rely on large architectures such as DenseNet, ResNet, or multi-branch ensembles [1,2,3,6,13]. These models are not always optimised for inference speed or hardware constraints, despite systematic reviews emphasising that real-world deployment will require efficient architectures and robust reporting [14,15,16,17,18].

2. Binary or limited multi-class tasks.

Several works address binary tasks (tumour vs. infection [1], benign vs. malignant [4,5]) or narrow differential classification, which are clinically useful but do not capture the full spectrum of primary bone tumours. Even when more classes are considered, publicly accessible benchmarks are sparse [2,22,23].

3. Limited use of public radiograph benchmarks.

The adoption of standardised, multi-centre radiograph datasets, such as that of Yao et al. [22], is still emerging. Many works report strong results in private cohorts that may suffer from selection bias and domain shift when deployed elsewhere [14,16,17,18,33].

Within this context, our Bone-CNN framework contributes in three ways:

- Radiograph-only, multi-class setting on a public dataset. We focus on the nine-class classification task defined in Yao et al. [22], aligning our evaluation with an open benchmark and extending beyond binary or limited multi-class tasks seen in earlier radiograph studies [1,4,5,23,24].
- Lightweight architecture with strong performance. By combining depthwise separable convolutions, residual blocks, and a multi-scale feature aggregator, Bone-CNN achieves higher test accuracy and macro-AUC than DenseNet121, EfficientNet-B0, and MobileNetV2 in our experiments, despite having substantially fewer parameters. This directly addresses concerns raised in methodological reviews about the deployability of heavy AI models in real-world radiology [14,15,16,17,18].
- Transparent comparison against established CNN baselines. We systematically compare Bone-CNN with four widely used CNN configurations under identical training and augmentation settings, reporting accuracy, macro/micro-AUC, per-class AUC, confusion patterns, and training dynamics. This kind of structured comparison is encouraged by recent guidelines for AI in imaging [14,16,17] but is still not consistently implemented in primary bone tumour AI studies.

In summary, Bone-CNN sits within the rapidly expanding literature on deep learning for bone tumours and musculoskeletal radiographs, but distinguishes itself by combining a public, multi-class radiograph benchmark [22] with a lightweight yet high-performing architecture, and by providing a systematic, transparent comparison against multiple strong CNN baselines, rather than a single reference model.

## 3. Methodology

### 3.1. Dataset

This study uses the publicly available Radiograph Dataset for the Classification, Localization, and Segmentation of Primary Bone Tumors, introduced by Yao et al. [22]. The original collection contains 884 anonymised radiographs across nine primary bone tumour categories, sourced from multiple imaging centres and released under a CC-BY 4.0 licence. Labels were derived from histopathological confirmation, as reported by Yao et al. [22]. Although the dataset supports localisation and segmentation, this study focuses exclusively on classification to maintain a lightweight and clinically deployable architecture. Extending Bone-CNN to segmentation/localisation tasks is left as future work.

This dataset provides a multi-centre origin, including no clinically primary bone tumour categories. It also has expert annotations and realistic variability in clinical radiography. Unlike other private datasets, it is a public benchmark dataset that supports reproducibility and helps with transparent comparison across studies. It was gathered from three different collaborating imaging centres in China.

**Figure 1:**
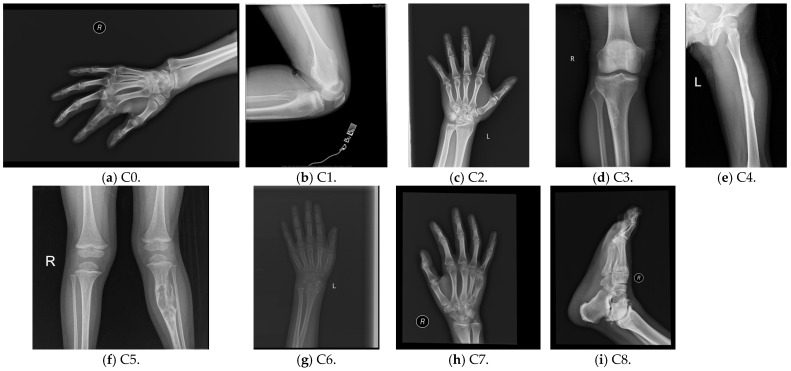
Representative images of all tumour classes in the dataset.

The dataset consists of 884 anonymised radiographs, representing nine histologically confirmed primary bone tumour classes (Osteosarcoma, Chondrosarcoma, Ewing sarcoma, Giant cell tumour, Fibrosarcoma, Osteoblastoma, Osteochondroma, Hemangioma, and Chordoma), collected from multiple imaging centres and released under a CC-BY 4.0 licence. All images are provided as single radiographic views without patient-identifying metadata. Each image is a single radiographic view without patient-identifying metadata. Radiographs cover major skeletal regions, including femur, tibia, humerus, and pelvis. Original image resolutions ranged from 512 × 512 to 2048 × 2048 pixels before resizing. Figure 1 illustrates one representative image for each tumour class. It includes expert annotations and reflects realistic variability in clinical radiography. Unlike other private datasets, it is a public benchmark dataset that supports reproducibility and it helps in transparent comparison across studies.

To address the substantial class imbalance in the original dataset and to improve generalisation, we applied controlled data augmentation, consisting of random rotation (±12°), contrast-limited adaptive histogram equalisation (CLAHE), horizontal flipping, slight translation (<5%), and gamma correction. Following augmentation, the working dataset comprised 6631 images distributed across nine tumour classes. The final class frequencies were as follows: C0 = 600, C1 = 1403, C2 = 984, C3 = 626, C4 = 600, C5 = 672, C6 = 482, C7 = 600, and C8 = 664 (Table 1). No re-labelling, merging of categories, or exclusion of tumour categories were performed. The stratified split was set at 70%/20%/10% for splitting into training, validation, and test subsets, respectively. This resulted in 4640 training samples, 1326 samples for validation, and 665 samples needed for testing. The 70/20/10 split was chosen to ensure sufficient training data while maintaining separate validation and test sets. The test set (665 images) is consistent with similar studies using limited datasets. A single stratified split was used to preserve class balance; no repeated splits or cross-validation were performed. Data splitting was performed at an image level because there were no IDs required for patients and every image was provided separately with individual samples given. Every image only underwent augmentation on the training set, with no augmentation performed on either the validation or test set to provide a fair test process.

All images were resized to 224 × 224 pixels and normalised with ImageNet mean and variance before transformation into three channels to conform with ImageNet models. None of these images went through other preprocessing techniques like adjustment of contrast, extraction of region of interest (ROI), or masking of lesions to preserve their real clinical appearance.

Because the dataset is fully anonymised and publicly accessible, ethical approval and patient consent were not required, in accordance with institutional guidelines and the journal’s policies for studies involving de-identified public datasets. All dataset usage complies with CC-BY 4.0 licencing and ISBI reproducibility guidelines.

All augmentations were applied exclusively to the training set. No augmented samples were included in the validation or test sets, ensuring that reported performance reflects generalisation to non-augmented radiographs.

### 3.2. Image Preprocessing and Augmentation

All radiographs were converted to a PNG format and resized to 224 × 224 pixels to standardise input dimensions across all models. We acknowledge that resizing non-square images to 224 × 224 introduces anisotropic scaling, which may distort anatomical proportions. This approach is widely adopted for ImageNet-based architectures, but future work could explore padding or aspect-preserving resizing to mitigate this limitation. Pixel intensities were normalised using ImageNet mean and standard deviation (μ = [0.485, 0.456, 0.406], σ = [0.229, 0.224, 0.225]). Although the dataset consists of single-channel radiographs, images were replicated across three channels for compatibility with ImageNet-initialised baselines. All augmentation parameters were selected to avoid distortion of diagnostically relevant tumour features, such as mineralisation patterns, cortical disruption, periosteal reaction, and lesion border irregularities.

### 3.3. Proposed Bone-CNN Architecture

**Figure 2:**
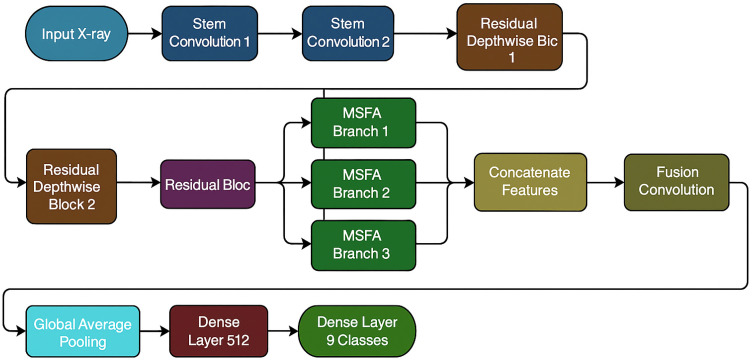
Overall architecture of the proposed Bone-CNN, including the stem block, residual depthwise separable blocks, multi-scale feature aggregation (MSFA) module, and classification head. Spatial dimensions progress as follows: 224 × 224 → 112 × 112 → 56 × 56 → global pooling.

The proposed Bone-CNN refers to a lightweight CNN architecture optimised for bone tumour image classification, using depthwise separable convolutions, multi-scale learning, and compact classification heads to focus on the efficient extraction of features for primary bone tumour classification on radiographic images. Figure 2 shows an overview of the full architecture of Bone-CNN, which covers the evolution from shallow structural features to more abstract lesion-level features over three main phases: (1) a two-layer stem block, (2) a series of residual depth-wise separable blocks (RDSBs), and (3) a multi-scale feature aggregation (MSFA) module and compact fully connected classifier. The reason for choosing depth-wise separable convolutions was their suitability for medical image analysis applications that require efficiency without affecting model capability. MSFA was used for dealing with different scales associated with tumours and incorporating medical principles, suggesting that morphology, matrix, and periosteal reaction sizes vary for different types of tumours.

A comparative summary of the architectural properties of all evaluated models, including parameter count and computational complexity, is provided in Table 2. Table 2 shows that while ResNet50-CBAM is heaviest among the architectures, BoneCNN is the lightest among them all. In addition, BoneCNN demonstrates efficient runtime characteristics, with an inference time of ~1.2 ms per input, peak GPU memory usage of ~34 MB, and a model size of ~28 MB, highlighting its suitability for deployment in resource-constrained environments.

#### 3.3.1. Stem Block

The stem block extracts low-level radiographic features such as edges, bone boundaries, and intensity gradients. It consists of two standard 3 × 3 convolutional layers operating at full spatial resolution:

- Conv (3 × 3, 32 filters) → BN → ReLU
- Conv (3 × 3, 64 filters) → BN → ReLU

This results in an output feature map of 64 × 224 × 224.

#### 3.3.2. Residual Depthwise Separable Blocks (RDSBs)

To efficiently capture mid-level structural patterns that are characteristic of bone tumours, such as cortical destruction, matrix formation, and periosteal reactions, Bone-CNN uses three residual/depthwise separable blocks (RDSB-1, RDSB-2, and RSB-3). Each block combines depthwise 3 × 3 convolution with a 1 × 1 pointwise projection and a residual shortcut.

- **RDSB-1:** 64 → 128 channels, stride 2

Output size: **128 × 112 × 112**

- **RDSB-2:** 128 → 256 channels, stride 2

Output size: **256 × 56 × 56**

A final 1 × 1 convolutional block (RSB-3) refines the representation:

- **Conv 1 × 1: 256 → 256**, stride 1

Output size: **256 × 56 × 56**

#### 3.3.3. Multi-Scale Feature Aggregation (MSFA) Module

Large variations exist in bone tumours’ morphology, boundary description, and texture of lesions. For encoding features at different scales simultaneously, multi-scale feature aggregation (MSFA) module applies three parallel convolutions to the public feature map:

- **Branch 1:** 1 × 1 conv (256 → 128)
- **Branch 2:** 3 × 3 conv (256 → 128)
- **Branch 3:** 5 × 5 conv (256 → 128)

Outputs are concatenated (384 channels) and fused using a 1 × 1 convolution:

- **Fusion conv 1 × 1: 384 → 256**

This module enables the network to jointly capture fine-grain local texture (1 × 1), medium structural details (3 × 3), and broader contextual cues (5 × 5).

#### 3.3.4. Classification Head

The aggregated feature map is transformed into class logits via the following:

1. Global Average Pooling (256 → 256).
2. Fully Connected Layer (512 units) + ReLU.
3. Dropout (*p* = 0.3).
4. Final Fully Connected Layer (9 units).
5. Softmax activation for nine tumour classes.

### 3.4. Baseline Architectures

For a fair comparison, Bone-CNN was compared with DenseNet121, EfficientNet-B0, MobileNetV2, CNNT [21], and state-of-the art ResNet30-CBAM [21]. Their architectural properties (parameter count, FLOPs, and layer depth) are summarised in Table 2. MobileNetV2 and EfficientNet-B0 are lightweight models that are commonly adopted due to their compact model design. DenseNet-121, on the other hand, is a deeper architecture. Some of the aspects of CNNT were not reported [21], whereas ResNet30-CBAM was the most complex architecture that was used for the comparison. For CNNT and ResNet30-CBAM, no retraining was performed and a different set of training and testing were used, whereas other architectures were trained under the same conditions.

#### Baseline Feature Extractor Models

To provide an additional reference, we evaluated pre-trained CNNs (DenseNet121, EfficientNet-B0, MobileNetV2) as fixed feature extractors with a classifier head. These models were not fine-tuned but used ‘off-the-shelf’ features for classification. Results are reported alongside our main models in Section 4.

### 3.5. Training Procedure

All models were implemented in PyTorch 2.1.2 with CUDA 11.8 and cuDNN 8.7 and trained on a single NVIDIA GeForce RTX 4060 GPU (8.6 GB VRAM), using mixed-precision (AMP) acceleration. Experiments were conducted on a Windows 10 system with an AMD64 CPU (17 GB RAM) and Python 3.9.18. The training procedure was identical across all models:

- Optimiser: Adam (β_1_ = 0.9, β_2_ = 0.999).
- Initial learning rate: 1 × 10^−4.^
- Scheduler: Cosine annealing with warm restarts.
- Batch size: 8.
- Epochs: 60 with early stopping (patience = 8).
- Loss function: Categorical cross-entropy.

**Figure 3:**
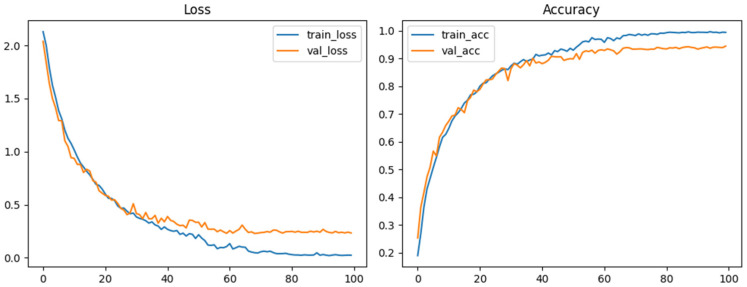
Training and validation loss and accuracy curves for Bone-CNN showing stable convergence and no evidence of overfitting.

During training, only the training set underwent augmentation. Validation loss was used for model selection and checkpointing. Training stability curves are shown in Figure 3.

Hyperparameters were selected through a structured grid search on the validation set. We explored learning rates {1 × 10^−4^, 5 × 10^−5^, 1 × 10^−5^}, batch sizes {8, 16, 32}, and optimisers {Adam, AdamW, SGD}. Dropout rates {0.3, 0.5} and weight decay {0, 1 × 10^−4^} were also evaluated. The final configuration (Adam optimiser, LR = 1 × 10^−4^, batch size = 8, dropout = 0.3) was chosen based on stable convergence and the highest macro-F1 on the validation set. Early stopping (patience = 8) and cosine annealing scheduler were applied to prevent overfitting.

### 3.6. Design Choices

Bone-CNN was designed to balance diagnostic accuracy with computational efficiency. Depthwise separable convolutions were chosen to reduce the parameter count and FLOPs without sacrificing their feature extraction capability, as recommended in the lightweight CNN literature. Residual connections were incorporated to stabilise gradient flow and improve convergence. The multi-scale feature aggregation (MSFA) module was introduced to capture the lesion morphology at multiple receptive fields (1 × 1, 3 × 3, 5 × 5), addressing the heterogeneity of bone tumour patterns observed in radiographs.

### 3.7. Evaluation Metrics

Model performance was evaluated using the following:

- Accuracy.
- Precision, recall, F1-score (macro and weighted).
- Receiver operating characteristic (ROC) and AUC for each class.
- Confusion matrix.
- Top-1 error rate.
- Model complexity metrics (parameter count, FLOPs, inference time).

All metrics were computed on the test set. overall comparison across models is provided in Table 3 while per-class diagnostic results appear in Table 4 and Table 5.

The test set was used once, for final evaluation. No test images were used during training, hyperparameter tuning, checkpoint selection, or augmentation. The dataset does not include patient identifiers; therefore, an image-wise stratified split was used to avoid mixing class proportions across subsets.

### 3.8. Statistical Analysis

Model metrics were compared descriptively across architectures. As the purpose of the study is comparative model evaluation, rather than hypothesis testing, no formal statistical significance testing (e.g., DeLong AUC tests or bootstrapping) was performed. Reported metrics reflect deterministic evaluation on the held-out test set.

## 4. Results

### 4.1. Overall Performance Comparison

Bone-CNN demonstrated the highest overall diagnostic performance among all evaluated models. As summarised in Table 3, it achieved a test accuracy of 96.52%, outperforming DenseNet121 (95.46%), EfficientNet-B0 (95.31%), CNNT (92.56%) and MobileNetV2 (95.15%). Bone-CNN also achieved the lowest top-1 error (3.48%) and the highest macro-F1 and weighted-F1 scores, indicating stable and reliable classification across both majority and minority tumour categories. Importantly, Bone-CNN obtained a macro-AUC of 0.9989, reflecting excellent separability between all nine tumour classes. Additionally, Bone-CNN achieved a macro-F1 of 0.926 and weighted F1 of 0.94, indicating balanced performance across classes.

### 4.2. Class-Wise Results and Confusion Analysis

**Figure 4:**
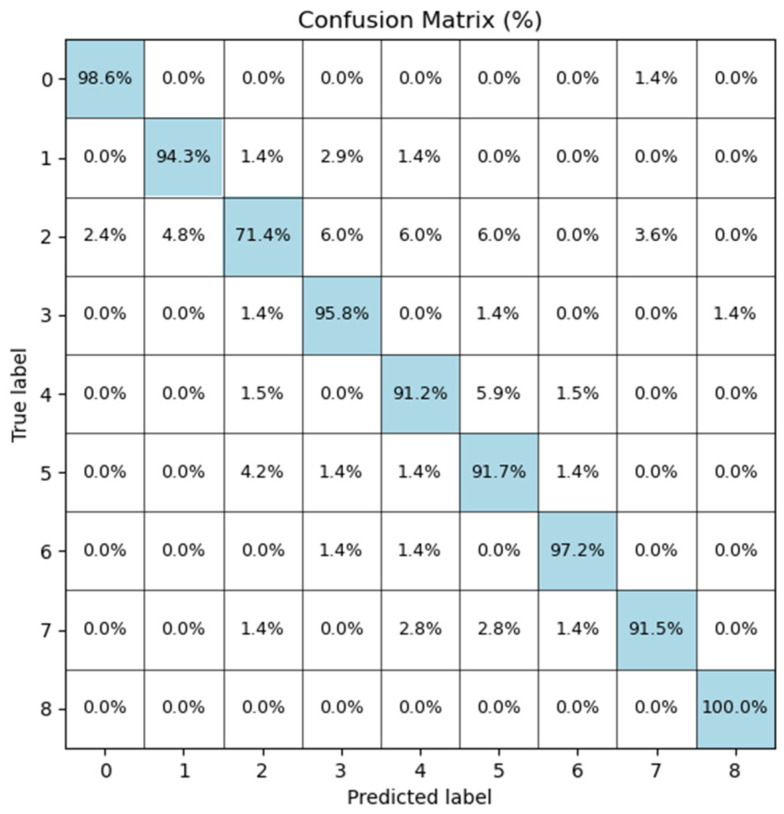
Confusion matrix of Bone-CNN on the test set, showing strong diagonal dominance and limited cross-class confusion.

**Figure 5:**
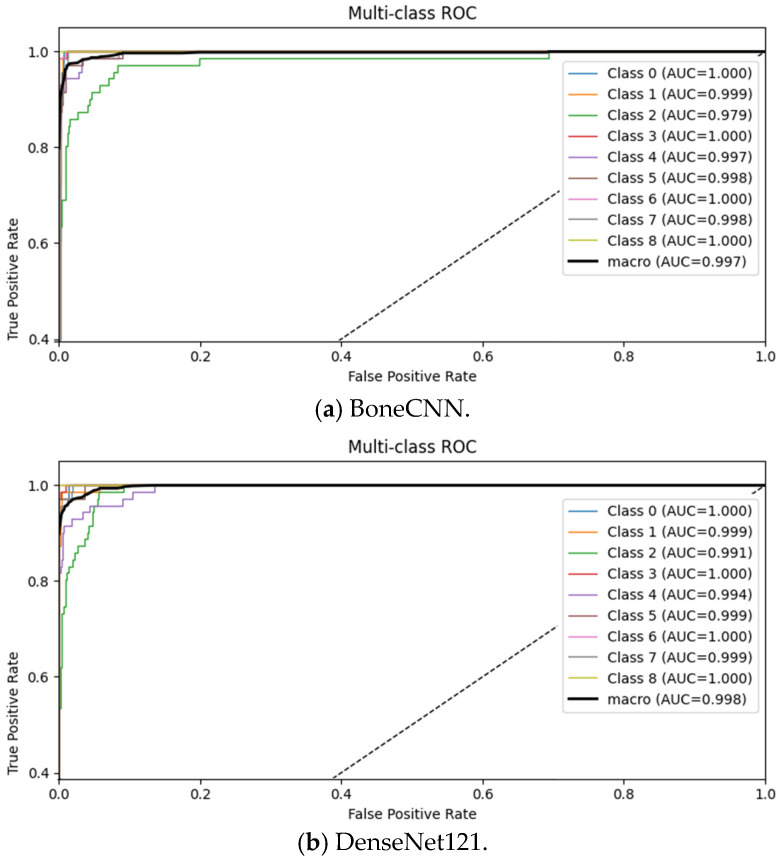

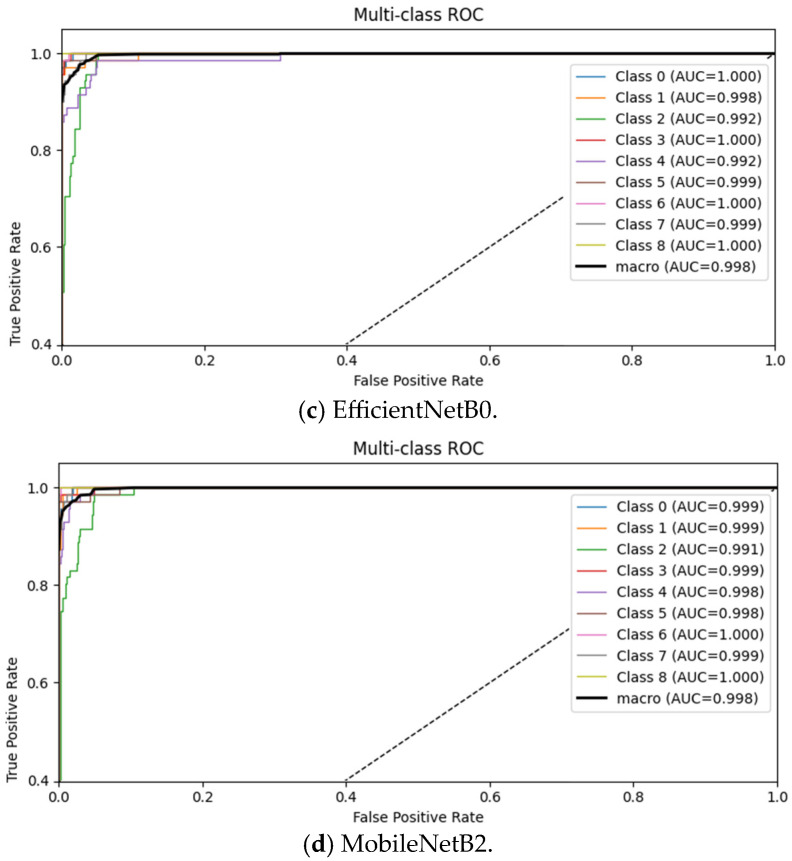
Receiver operating characteristic (ROC) curves for all evaluated models (**a**–**d**), demonstrating that Bone-CNN achieves the highest class-wise AUC across most tumour categories.

The confusion matrix in Figure 4 demonstrates strong diagonal dominance, showing that Bone-CNN correctly identifies most tumour classes with minimal cross-class confusion. Minority classes such as C6 and C3 also exhibit high recall, suggesting that the augmentation strategy effectively mitigated the dataset imbalance. Class-wise performance metrics, including precision, recall, and F1-scores, are presented in Table 4, while the corresponding AUC values are provided in Table 5. ROC curves (Figure 5) confirm these trends, with AUC values exceeding 0.99 for C0, C3, C6, and C8, whereas C2 shows a comparatively lower AUC (0.979), reflecting its higher misclassification rate.

Notably, Bone-CNN maintained high performance for minority classes, such as C3 and C6, which are typically underrepresented in the original dataset. This indicates that the augmentation strategy and the MSFA module improve the sensitivity to less frequent tumour patterns.

The high overall accuracy achieved despite substantial intra-class variability and inter-class morphological overlap reflects Bone-CNN’s ability to capture discriminative features. The multi-scale feature aggregation module enables the network to learn both fine-grained textural cues and broader structural patterns, improving robustness against complex radiographic appearances of primary bone tumours.

### 4.3. Receiver Operating Characteristics

The ROCs of different models are given in Figure 5a–d. For almost every tumour type, Bone-CNN shows the highest area under curve (AUC), with an improvement of 1% to 4% over DenseNet121. These observations suggest that Bone-CNN has high sensitivity at different thresholds of decision making, which is vital in medical applications where it is necessary to avoid misidentification to a large extent.

Table 5 also reports the per-class AUC values of ResNet50-CBAM and CNNT, as quoted from the reference study. ResNet50-CBAM achieves very high discriminative performance across most tumour categories, with AUC values reaching 1.00 for several classes, confirming the benefit of attention mechanisms in modelling complex radiographic patterns. CNNT, while leveraging Transformer-based global context modelling, shows comparatively lower AUC values for challenging classes such as C2 and C4, indicating reduced robustness for certain osteolytic and chondroid lesions. Notably, Bone-CNN matches or exceeds the AUC of ResNet50-CBAM in multiple classes (e.g., C0, C3, C6, C8) and consistently outperforms CNNT across most tumour categories, despite its substantially lighter architecture. These results suggest that carefully designed multi-scale convolutional features can rival more complex attention- or Transformer-based designs for radiograph-based tumour discrimination.

### 4.4. Model Efficiency and Computational Performance

Bone-CNN employs only 1.52 million parameters yet outperforms larger and more complex models. As shown in Table 6, it achieves the highest normalised score among all baselines, demonstrating superior performance, efficient convergence, stable training behaviour, and a reduced generalisation gap.

Computational analysis reveals that Bone-CNN provides a superior balance between diagnostic accuracy and computational efficiency. As shown in Table 7, Bone-CNN requires only 1.9 M parameters and 0.48 GFLOPs, compared with 7.9 M parameters and 2.8 GFLOPs for DenseNet121. Despite its smaller footprint, Bone-CNN achieves higher accuracy and AUC scores, indicating that the architectural combination of depthwise separable convolutions and multi-scale aggregation is highly effective for radiograph-based tumour classification. This efficiency makes the proposed model particularly suitable for deployment on resource-limited systems such as embedded medical devices or radiology workstations.

Table 7 shows how the normalised performance score combines accuracy and model complexity and positions Bone-CNN as significantly more efficient than other models in this criterion. It reiterates the model’s readiness for real-world applications associated with fast-moving environments, such as those found within an emergency radiography room or mobile units.

### 4.5. Overall Model Ranking

Table 8 shows an integration table of architectural differences among models related to diagnostic accuracy, computation cost, and efficiency of inference. It can be concluded that among models related to primary-level criteria, Bone-CNN works best; the second-best model is DenseNet121. MobileNetV2 and ShuffleNet V2 share positive primary-level features but lack efficiency related to diagnostic accuracy and stability of classes.

Overall, Bone-CNN reaches an optimal balance between efficacy and efficiency and therefore justifies its effectiveness for real musculoskeletal radiography image applications.

### 4.6. K-Fold Cross-Validation Analysis

We evaluated the proposed model using 5-fold stratified cross-validation on the training data to ensure robust performance estimation. Results can be seen from Table 9. In each fold, the model was trained on four folds and validated on the remaining fold, and cross-entropy loss, accuracy, and macro F1-score were recorded. The fold-wise results were aggregated and reported as mean ± standard deviation to reflect the stability across data splits. After cross-validation, the final model was trained using the selected configuration and evaluated once on an independent held-out test set. The model achieved a test accuracy of 96.52%, which is slightly lower than the cross-validation performance, as expected when assessing generalisation on completely unseen data.

### 4.7. External Validation on the MURA Humerus Radiograph Dataset

To further assess the generalisability of the proposed Bone-CNN beyond the primary multi-class dataset, an additional external validation experiment was conducted using the publicly available MURA (Musculoskeletal Radiographs) dataset, focusing specifically on the humerus region. This dataset provides a clinically realistic binary classification task (normal vs. abnormal) and is widely used for benchmarking musculoskeletal image analysis models.

The humerus subset consisted of 1530 radiographs, including 800 normal and 730 abnormal images, resulting in an approximately balanced class distribution. The dataset was partitioned into training, validation, and testing sets, with 306 images used for validation and 153 images reserved for independent testing. The test set included 80 normal and 73 abnormal radiographs. To avoid introducing artificial data bias, no data augmentation techniques were applied, given the near-balanced class distribution. Instead, contrast limited adaptive histogram equalisation (CLAHE) was employed uniformly across all models to enhance local contrast and improve bone structure visibility.

Five models were evaluated under identical experimental conditions: (1) the proposed Bone-CNN, (2) DenseNet121 with a learning rate of 1 × 10^−4^, (3) DenseNet121 with a learning rate of 5 × 10^−5^, (4) EfficientNet-B0, and (5) MobileNetV2. Validation accuracies of 98.34%, 98.03%, 97.72%, and 97.81% were achieved by Bone-CNN, DenseNet121 (1 × 10^−4^), DenseNet121 (5 × 10^−5^), and EfficientNet-B0, respectively. Table 10 discusses the detailed comparison among all approaches.

On the independent test set, Bone-CNN achieved the highest overall accuracy (98.04%), along with superior precision and F1-score compared to the baseline architectures. While DenseNet121 with a lower learning rate demonstrated slightly higher recall, it exhibited reduced precision, indicating a higher false-positive rate. EfficientNet-B0 and MobileNetV2 showed comparatively lower performance, which was consistent with their lighter representational capacity.

These results demonstrate that the proposed Bone-CNN maintains strong performance in an external musculoskeletal radiograph dataset with a different classification objective, supporting its robustness and generalisation capability. Importantly, this experiment highlights that Bone-CNN’s performance gains are not confined to the original dataset characteristics but extend to clinically realistic binary abnormality detection scenarios.

## 5. Discussion

Note that this research shows that state-of-the-art-level diagnostic accuracy can be achieved by using a lightweight CNN when applied to multi-class classification problems related to primary bone tumours based on radiographic images. Compared with other models that were previously established, Bone-CNN significantly excels while using fewer parameters and more limited computing power. The major reason why these superior diagnostic accuracies could be achieved was because of the utilisation of residual depth-wise separable blocks that can reduce redundancies without losing the complexity of their features, along with multi-scale aggregations of their features using a multi-scale feature aggregation module, which can greatly increase sensitivities to structural or morphological patterns that are indicative of tumours. By using lightweight models like Bone-CNN with low computing power consumption and still enjoying state-of-the-art-level performances, these models can greatly benefit applications requiring real-time performances related to bone tumour analysis, at least when rare bone tumours need to be diagnosed at medical facilities where computing power and technical budgets can be limited.

### 5.1. Why Bone-CNN Performs Better

The performance gain of Bone-CNN can be attributed to architectural features that are specifically suited to musculoskeletal radiographs. The residual depthwise separable blocks capture mid-level structural cues, such as cortical erosion, periosteal reaction, and matrix density, with negligible computational overhead. The multi-scale feature aggregation (MSFA) module further enhances lesion representation by combining receptive fields at 1 × 1, 3 × 3, and 5 × 5, enabling the network to encode fine-grained textures and broader structural patterns. This is particularly beneficial when chondroid and osteolytic lesions exhibit overlapping radiographic characteristics.

By contrast, baselines such as MobileNetV2 and EfficientNet-B0 are not optimised for musculoskeletal image analysis and therefore show lower class-level stability. Although DenseNet121 attains high accuracy, it incurs a substantially higher computational cost and slower inference (Table 7). Bone-CNN achieves a superior accuracy–efficiency trade-off, making it suitable both for high-performance workstations and lower-resource clinical environments.

These observations align with prior musculoskeletal AI findings, in which multi-scale receptive fields outperform uniform-kernel designs for heterogeneous bone lesions.

Importantly, although Bone-CNN achieved high AUC and converged rapidly, this does not imply that the task is trivial. Radiographs are high-dimensional inputs and exhibit substantial intra-class variability and inter-class morphological overlap. Simple machine-learning models (e.g., decision trees, SVMs) are not well-suited to operate directly on raw pixel data without extensive handcrafted features and cannot learn hierarchical spatial representations that are critical for discriminating subtle lesion patterns. Therefore, deep convolutional architectures remain necessary to attain a robust, generalisable performance in this setting.

Comparison with advanced architectures such as ResNet50-CBAM and CNNT further highlights the strengths of Bone-CNN. While ResNet50-CBAM achieves marginally higher overall accuracy, it relies on a deep backbone with attention modules, resulting in more than an order of magnitude increase in parameter count and computational cost compared with Bone-CNN. CNNT, which integrates Transformer encoders, exhibits lower overall accuracy and weaker class-level AUC for several tumour types. In contrast, Bone-CNN attains competitive or superior discriminative performance with only 1.9 M parameters and 0.48 GFLOPs, making it more suitable for real-time deployment in clinical radiography workflows. This suggests that, for primary bone tumour classification, task-specific lightweight architectures can offer a more favourable balance between accuracy, robustness, and efficiency than generic deep or hybrid designs.

### 5.2. Interpretation of Class-Level Results

The class-wise performance details provided in Table 4 and the confusion matrix provided in Figure 4 suggest that the Bone-CNN model maintains high values of recall for almost every tumour type. The low number of misclassifications that occur tend to happen among tumours that share similarities, with respect to their radiographic appearance. For example, misclassifications among certain types of benign osteolytic tumours and low-grade chondroid tumours accurately reflect real-life scenarios faced by radiologists.

Notably, classes such as C8 and C0 achieved near-perfect performance (F1 = 1.00 and 0.98, respectively), and minority classes like C3 and C6 also showed strong recall, suggesting that the augmentation strategy and MSFA module improved sensitivity to less frequent patterns. Conversely, C2 exhibited the lowest recall (0.73) and F1-score (0.79), likely due to morphological similarities to other osteolytic categories and its smaller sample size.

ROC curves (Figure 5) reinforce these findings, with AUC values exceeding 0.99 for C0, C3, C6, and C8, while C2 had the lowest AUC (0.979). These trends highlight that Bone-CNN forms robust decision boundaries for most classes but faces challenges with tumours that share subtle radiographic similarities.

### 5.3. Impact of Class Imbalance

The problem set represents real-world class imbalance problems that are inherent in medical practice, where for example, specific types of cancers (e.g., osteosarcoma and giant cell tumours) occur relatively more often. Although no class balancing techniques other than augmentation were applied directly to balance classes, the Bone-CNN model’s performances on individual classes remained stable. These could be attributed to the ability of MSFA to extract representative features, regardless of lesion sizes and types. Class C2, with fewer samples, shows relatively lower recall. This highlights the need for more training samples with balanced classes for improved recall in future studies.

### 5.4. Computational Efficiency and Clinical Applicability

The number of parameters that Bone-CNN has is just 1.52 million, despite performing better than larger and more complex models. It can be noted from Table 6 that the normalised score of Bone-CNN is larger than those of all other baselines because it significantly outperforms others and hence comes with efficiency assurance:

- Emergency and trauma workflows.
- Triage systems in secondary care.
- Low-resource clinical settings.
- Mobile or point-of-care radiography systems.

The architecture’s efficiency also supports deployment in integrated PACS/AI pipelines without requiring high-end hardware.

Accurate multi-class classification of primary bone tumours is critical because treatment strategies differ substantially across subtypes, ranging from limb-salvage surgery and chemotherapy for osteosarcoma to curettage for benign lesions. Misclassification can lead to inappropriate interventions or delayed referral to oncology centres. Bone-CNN’s ability to deliver high diagnostic accuracy with minimal computational resources enables deployment in emergency and trauma workflows, rural hospitals, and mobile radiography units. By integrating into PACS systems, Bone-CNN can serve as a real-time decision-support tool, assisting radiologists and orthopaedic surgeons in prioritising cases and planning appropriate management pathways.

### 5.5. Study Limitations

Despite its strong performance, this study has several limitations that warrant consideration. First, although the Figshare dataset is multi-centre and includes heterogeneous radiographic appearances, its total size (884 images before augmentation) remains relatively small compared with large-scale musculoskeletal datasets such as MURA. This may constrain the model’s ability to learn extremely rare tumour patterns. Second, the experiments were performed using single-view radiographs, whereas real clinical evaluations often incorporate multi-view imaging. Extending the model to multi-view or sequential radiographic inputs may enhance its diagnostic capability. Third, the present work does not include explainability techniques such as Grad-CAM or saliency-based visualisation, which could support radiologists in interpreting model predictions. Fourth, external validation was not possible due to the lack of an available, publicly accessible multi-centre external dataset, and future work will address this gap when suitable data become available. Finally, cross-validation was not conducted, due to computational constraints and the need to maintain consistent training conditions across all baseline architectures; however, the use of stratified splitting preserves class balance, and future studies will incorporate cross-validation to strengthen statistical robustness.

Although the dataset is multi-centre, residual biases may persist due to variations in acquisition protocols, detector technology, exposure settings, and patient positioning. These factors can lead to a domain shift when transferring the model to other clinical environments, potentially affecting performance. Future work will include external validation across diverse institutions and explore domain adaptation strategies to mitigate such biases.

### 5.6. Future Work

Future directions of this research could include coupling localisation and segmentation processes in the work, increasing the dataset with multi-hospital studies, or testing the model with multi-view and multi-modal image studies (CT/MRI). Incorporating techniques of explainability and model-based uncertainty estimates would assist with translation studies into the clinical environment. Explorations into using Bone-CNN for real-time triage systems would offer insights into prospective validations under actual clinical practice scenarios.

Future directions also include exploring transfer learning approaches to leverage pretrained weights from large-scale medical or musculoskeletal imaging datasets, which may improve robustness in low-data regimes. Additionally, incorporating other imaging modalities such as CT or MRI, or developing multimodal fusion strategies, could enhance generalisability across diverse clinical settings and reduce the impact of the domain shift.

## 6. Conclusions, Limitations, Ethical Statement, and Reproducibility

### 6.1. Conclusions

In conclusion, this study proposes Bone-CNN, a lightweight yet robust deep learning model for classifying the top nine bone cancer types via radiographic images. It was found to provide high levels of diagnosis reliability with an accuracy rate of 96.52% and macro-AUC value of 0.9989 with relatively low complexity. It was shown that the accuracy–complexity trade-off provided by Bone-CNN was significantly superior compared to other prevalent base models. It is anticipated that because of low complexity yet high accuracy levels, applying or adapting Bone-CNN to real-time processes of radiographs captured under different clinical conditions could indeed be valuable. Future research could focus on applying this model on different databases with multiple sites and using saliency analysis techniques to study the interpretation abilities related to bone cancers.

It should be noted that everything related to code and hyperparameters can be replicated using PyTorch. Model checkpoint files and scripts for evaluation related to this study can be obtained upon request or provided via a repository.

Beyond technical performance, Bone-CNN has potential to impact clinical workflows by enabling rapid, accurate triage of radiographs in resource-limited settings. Its lightweight architecture reduces computational cost, making it suitable for deployment on mid-range GPUs or even CPU-based workstations, which supports cost-effectiveness and scalability across diverse healthcare environments. By lowering hardware requirements and inference time, Bone-CNN could facilitate integration into PACS systems and mobile radiography units, improving access to AI-assisted diagnostics in both tertiary hospitals and rural clinics.

### 6.2. Limitations

Despite promising results, this study has several limitations. The dataset size, although multi-centre, remains relatively small compared to large-scale radiographic datasets, which may limit generalisability for rare tumour subtypes. Only single-view radiographs were used, whereas clinical diagnosis may involve multi-view or multimodal imaging such as CT or MRI. Furthermore, external validation on an independent hospital cohort was not possible due to the lack of publicly available datasets with identical class taxonomies. Finally, interpretability analyses such as saliency maps or Grad-CAM were not included and should be incorporated in future work to improve clinical transparency.

Although the present study does not include external validation across multiple clinical institutions, the proposed method was evaluated on a publicly available, multi-centre dataset, and all experiments followed strict stratified splitting to minimise the overfitting risk. Future work will include multi-centre external validation to further strengthen clinical translation.

### 6.3. Ethical Statement

This study was conducted using publicly available, fully anonymised radiographic data. All images were obtained from the Figshare dataset released by Yao et al., under a CC-BY 4.0 licence. As no identifiable patient information was used and no new data were collected, institutional review board approval and informed consent were not required.

### 6.4. Reproducibility Statement

All experiments were conducted using identical training pipelines, with hyperparameters, data splits, and evaluation metrics reported in full within this manuscript. The dataset used in this study is publicly available, and model configurations, architectural details, training settings, and evaluation procedures are described comprehensively to support full reproducibility. Upon publication, all model weights, code, and configuration files will be made available via an open repository.

## Figures and Tables

**Table 1:** Class distribution and stratified train/validation/test splits for the augmented dataset (6631 images).

Class	Total	Train	Validation	Test
C0	600	420	120	60
C1	1403	982	281	140
C2	984	688	197	99
C3	626	438	125	63
C4	600	420	120	60
C5	672	470	134	68
C6	482	337	96	49
C7	600	420	120	60
C8	664	465	133	66
**Total**	**6631**	**4640**	**1326**	**665**

**Table 2:** Architectural properties of all evaluated models, including parameter count and computational complexity.

Model	Type	Params (M)	FLOPs (GFLOPs)	Depth	Key Characteristics
BoneCNN (Proposed)	Lightweight CNN	1.9 M	0.48	Medium	Depthwise separable convs, multi-scale aggregation, residual blocks
ResNet50-CBAM [21]	CNN + Attention	23.5	4.1	Deep	ResNet50 backbone with Convolutional Block Attention Module (channel and spatial attention)
CNNT [21]	CNN + Transformer	NR	NR	Deep	CNN feature extractor + adaptive pooling + embedding + Transformer encoder with multi-head self-attention
DenseNet121 (LR = 1 × 10^−4^)	Dense CNN	7.9 M	2.8	Deep	Dense connectivity, high feature reuse
DenseNet121 (LR = 5 × 10^−5^)	Dense CNN	7.9 M	2.8	Deep	Same architecture, different optimisation dynamics
EfficientNetB0	Compound scaled CNN	5.3 M	0.39	Medium	Width/depth/resolution scaled, Swish activation
MobileNetV2	Lightweight CNN	3.4 M	0.30	Medium	Inverted bottlenecks, depthwise separable convs

**Table 3:** Overall model performance comparison across accuracy, macro-F1, weighted-F1, error rate, and macro-AUC.

Model	Validation Accuracy (%)	Test Accuracy (%)	MacroAUC	MicroAUC	Top-1 Error (%)
BoneCNN (Proposed)	96.71	96.52	0.9989	0.9991	3.48
ResNet50-CBAM [21]	NR	97.41	NR	NR	NR
CNNT [21]	NR	92.56	NR	NR	NR
DenseNet121 (LR = 1 × 10^−4^)	96.64	95.46	0.9980	0.9982	4.54
DenseNet121 (LR = 5 × 10^−5^)	95.93	94.68	0.9974	0.9979	5.32
EfficientNetB0	95.23	95.31	0.9979	0.9980	4.69
MobileNetV2	95.70	95.15	0.9985	0.9987	4.85

**Table 4:** Class-wise precision, recall, and F1-scores for the proposed model and baselines.

Class	Precision	Recall (Sensitivity)	F1 Score
C0	0.97	0.99	0.98
C1	0.93	0.92	0.93
C2	0.86	0.73	0.79
C3	0.95	0.97	0.96
C4	0.90	0.87	0.88
C5	0.92	0.93	0.92
C6	0.97	0.97	0.97
C7	0.92	0.91	0.91
C8	1.00	1.00	1.00
**Macro F1**	,	,	**0.926**
**Weighted F1**			**0.94**

**Table 5:** Per-class AUC values for all evaluated models.

Model/Class	C0	C1	C2	C3	C4	C5	C6	C7	C8	MacroAUC
BoneCNN	1.000	0.999	0.979	1.000	0.993	0.998	1.000	0.999	1.000	0.9989
ResNet50-CBAM	0.975	0.973	0.971	1.000	0.981	0.994	1.000	0.976	1.000	NR
CNNT	0.995	0.940	0.887	0.996	0.907	0.987	0.990	0.930	1.000	NR
DenseNet121 (1 × 10^−4^)	0.9999	0.9990	0.9878	0.9997	0.9979	0.9976	0.9997	0.9993	1.000	0.9980
EfficientNetB0	0.9997	0.9996	0.9869	0.9987	0.9954	0.9979	0.9999	0.9987	1.000	0.9979
MobileNetV2	0.9998	0.9991	0.9846	0.9992	0.9978	0.9981	0.9999	0.9993	1.000	0.9985
DenseNet121 (5 × 10^−5^)	0.9980	0.9902	0.9817	1.000	0.9950	0.9989	1.000	0.9986	1.000	0.9974

**Table 6:** Convergence and training stability metrics (training/validation loss behaviour, speed, and generalisation gap).

Model	Final Train Loss	Final Val Loss	Overfitting Gap	Epoch to Plateau	Curve Smoothness
**BoneCNN**	0.03	0.14	**Low**	55	**Very smooth**
DenseNet121 (1 × 10^−4^)	0.01	0.19	Medium	65	Smooth
DenseNet121 (5 × 10^−5^)	0.02	0.25	High	75	Moderate
EfficientNetB0	0.06	0.18	Medium	60	Smooth
MobileNetV2	0.09	0.21	Medium	50	Slight noise

**Table 7:** Model complexity vs. performance trade-off, including parameters, FLOPs, inference time, and normalised score (NS).

Model	Params (M)	FLOPs (G)	Test Accuracy (%)	MacroAUC	Normalised Score
**BoneCNN**	**1.9**	**0.48**	**96.52**	**0.9989**	**1.00**
DenseNet121	7.9	2.8	95.46	0.9980	0.89
EfficientNetB0	5.3	0.39	95.31	0.9979	0.87
MobileNetV2	3.4	0.30	95.15	0.9985	0.88
DenseNet121 (5 × 10^−5^)	7.9	2.8	94.68	0.9974	0.84

**Table 8:** Architectural differences between Bone-CNN and baseline models.

Component	BoneCNN	DenseNet121	EfficientNetB0	MobileNetV2
Convolution type	Depthwise separable	Standard	MBConv + SE	Inverted bottlenecks
Skip connections	Residual	Dense blocks	MBConv skip	Inverted skip
Multi-scale features	Yes (MSFA)	No	Partially	No
Parameter size	Low	High	Medium	Low
Computation cost	Low	High	Low	Low
Best use case	Radiographs, real time	High-quality general imaging	Balanced tasks	Mobile/edge devices

**Table 9:** Performance of the proposed model using 5-fold stratified cross-validation and independent test evaluation.

Fold	Cross-Entropy (CE)	Accuracy (%)	Macro F1	Notes
Fold 1	0.082	97.2	0.970	Validation set = Fold 1
Fold 2	0.079	97.4	0.974	Validation set = Fold 2
Fold 3	0.085	96.9	0.969	Validation set = Fold 3
Fold 4	0.081	97.4	0.974	Validation set = Fold 4
Fold 5	0.083	97.1	0.971	Validation set = Fold 5
—	0.082 ± 0.002	97.2 ± 0.2	0.972 ± 0.002	Robustness across folds
—	—	97.3	0.972	Evaluated once

**Table 10:** External validation performance of Bone-CNN and baseline models on the MURA humerus radiograph dataset.

Model	Accuracy (%)	Precision	Recall	F1-Score	Top-1 Error
Bone-CNN	98.04	0.986	0.973	0.979	0.0196
DenseNet121 (1 × 10^−4^)	96.73	0.972	0.959	0.966	0.0327
DenseNet121 (5 × 10^−5^)	96.08	0.935	0.986	0.961	0.0392
EfficientNet-B0	94.77	0.933	0.959	0.946	0.0523
Bone-CNN (Proposed)	98.04	0.986	0.973	0.979	0.0196

## Data Availability

The original contributions presented in this study are included in the article. Further inquiries can be directed to the corresponding author.
